# Patient perspectives on clinicians’ use of ambient AI scribes

**DOI:** 10.1093/jamiaopen/ooag104

**Published:** 2026-06-12

**Authors:** Shreya J Shah, Kirsten Naumann Murtagh, Danton Char, Yejin Jeong, Trevor Crowell, Margaret Smith, Stephen P Ma, April S Liang, Bethel Mieso, Clarissa Delahaie, Caroline Hsia, Nick Waggoner, Tait Shanafelt, Alpa Vyas, Joshua Frost, Mystique Smith-Bentley, Michael A Pfeffer, Christopher Sharp, Steven Lin, Patricia Garcia

**Affiliations:** Department of Medicine, Stanford University School of Medicine, Stanford, CA, 94305, United States; Division of Primary Care and Population Health, Stanford University School of Medicine, Stanford Healthcare AI Applied Research Team, Stanford, CA, 94305, United States; Division of Primary Care and Population Health, Stanford University School of Medicine, Stanford Healthcare AI Applied Research Team, Stanford, CA, 94305, United States; Department of Anesthesia, Stanford University School of Medicine, Stanford, CA, 94305, United States; Division of Primary Care and Population Health, Stanford University School of Medicine, Stanford Healthcare AI Applied Research Team, Stanford, CA, 94305, United States; Division of Primary Care and Population Health, Stanford University School of Medicine, Stanford Healthcare AI Applied Research Team, Stanford, CA, 94305, United States; Division of Primary Care and Population Health, Stanford University School of Medicine, Stanford Healthcare AI Applied Research Team, Stanford, CA, 94305, United States; Department of Medicine, Stanford University School of Medicine, Stanford, CA, 94305, United States; Department of Medicine, Stanford University School of Medicine, Stanford, CA, 94305, United States; Department of Medicine, Stanford University School of Medicine, Stanford, CA, 94305, United States; Technology and Digital Solutions, Stanford Medicine, Stanford, CA, 94305, United States; Technology and Digital Solutions, Stanford Medicine, Stanford, CA, 94305, United States; Technology and Digital Solutions, Stanford Medicine, Stanford, CA, 94305, United States; Department of Medicine, Stanford University School of Medicine, Stanford, CA, 94305, United States; Office of Patient Experience, Stanford Health Care, Palo Alto, CA, 94305, United States; Office of Patient Experience, Stanford Health Care, Palo Alto, CA, 94305, United States; Office of Patient Experience, Stanford Health Care, Palo Alto, CA, 94305, United States; Department of Medicine, Stanford University School of Medicine, Stanford, CA, 94305, United States; Technology and Digital Solutions, Stanford Medicine, Stanford, CA, 94305, United States; Department of Medicine, Stanford University School of Medicine, Stanford, CA, 94305, United States; Department of Medicine, Stanford University School of Medicine, Stanford, CA, 94305, United States; Division of Primary Care and Population Health, Stanford University School of Medicine, Stanford Healthcare AI Applied Research Team, Stanford, CA, 94305, United States; Department of Medicine, Stanford University School of Medicine, Stanford, CA, 94305, United States

**Keywords:** Artificial intelligence, ambient intelligence, generative artificial intelligence, large language models, electronic health records, informatics, documentation, ambient scribes

## Abstract

**Objective:**

This study assessed over 2000 patient perspectives on the use of ambient AI scribes in outpatient visits.

**Materials and Methods:**

This prospective quality improvement study was conducted at Stanford Health Care between May and July 2025. Outcome measures included patient perceived helpfulness of ambient AI scribes and patient interest in future use.

**Results:**

Among 2202 survey respondents, 70.1% patients found the ambient AI scribe helpful and 73.6% preferred future use of ambient AI scribes. Small but statistically significant differences were observed across gender, age, and race.

**Discussion:**

In an evaluation of patient perceptions of an ambient AI scribe integrated into clinical practice, the majority of patients who had experienced the technology found it acceptable, and most found the tool helpful and desired use with future visits.

**Conclusion:**

These results suggest that ambient AI scribes may be acceptable to many patients in clinical practice. Further research is needed to inform patient-centered design and workflow improvements.

## Background and significance

Ambient artificial intelligence (AI) scribes represent one of the fastest-growing applications of generative AI in healthcare. Prior studies suggest these tools can reduce clinician documentation burden, cognitive load, and burnout,^1–5^ but patient perspectives remain largely unknown. Existing data indicates that patients have neutral or favorable perceptions on visit quality, communication and overall care,^5^ but broader understanding is needed to guide successful patient-centered integration of these technologies.

## Objective

This study assessed over 2000 patient perspectives on the use of ambient AI scribes in outpatient visits to understand perceived helpfulness of ambient AI scribes, interest in future use, and overall interest in new technology. Secondary analyses analyzed potential differences in patient perspectives across gender, age, race, and visit type.

## Materials and methods

### Study design and setting

This prospective quality improvement study was conducted at Stanford Health Care (SHC) between May and July 2025 and followed SQUIRE reporting guidelines.

### Data collection and study measures

English-speaking adult patients who had a visit in which an ambient AI scribe was used received a post-visit survey through SHC patient experience emails. Ambient AI scribe questions were included as supplemental items within a routine post-visit patient experience survey. Clinicians were required to notify patients and others present in the room that the visit would be recorded before activating the ambient AI scribe tool. Patients could decline use of the ambient AI scribe tool. Clinicians then reviewed and edited the AI-generated draft note; final notes were made available for patients to review via a patient portal. Survey items, developed using the RE-AIM/PRISM framework in collaboration with the Office of Patient Experience, assessed perceived helpfulness of ambient AI scribes, interest in future use, and overall interest in new technology. Survey logic ensured patients received one survey.

### Statistical analysis

Analyses were conducted using SAS 9.4. Descriptive statistics summarized responses, and chi-square tests examined demographic differences. In descriptive analyses, missing responses were retained as a separate category in the denominator. In chi-square analyses, cases with missing data on either variable were excluded from the relevant comparison.

### Ethical approval

The Stanford University institutional review board determined the project met criteria for quality improvement and was exempt from informed consent.

## Results

2202 patients completed the survey (Table 1), including 1280 (58.1%) after primary care visits and 922 (41.9%) after specialty visits. Most respondents found the ambient AI scribe helpful (1543, 70.1%), preferred future use (1620, 73.6%), and expressed interest in new technology (1884, 85.6%) (Table 2).

**Table 1.** Patient demographics.

Demographic	Count *N* (%)
Gender	
Female	1241 (56.4%)
Male	951 (43.2%)
Missing/Other	10 (0.45%)
Race and Ethnicity	
White	1477 (67.1%)
Asian	348 (15.8%)
Other/Unknown	239 (10.9%)
Black/African American	90 (4.1%)
Missing/Declines to state	48 (2.2%)
Hispanic/Latino Status	
Non-Hispanic/Non-Latino	1893(86.0%)
Hispanic/Latino	161 (7.3%)
Missing/other/declines to state	148 (6.7)
Age	
<30	33 (1.5%)
30-39	77 (3.5%)
40-49	101 (4.6%)
50-59	197 (9.0%)
60-69	478 (21.7%)
70+	1316 (59.8%)
Visit type	
Primary Care	1280 (58.1%)
Specialty	922 (41.9%)

**Table 2.** Patient survey responses.

Survey question	Response option	Count *N* (%)
In the future, if you were to have another visit with this provider, would you like the provider to use the secure recording AI tool or would you not like that?		
	Would like provider to use AI scribe in future	1620 (73.6%)
	Would not like	416 (18.9%)
	Missing	166 (7.5%)
How helpful was it to have the secure recording AI tool active during your visit?		
	Found helpful	1,543 (70.1%)
	Did Not Find Helpful/Slightly Helpful	470 (21.3%)
	Missing	189 (8.6%)
How interested are you in using new technology?		
	Interested	1,884 (85.6%)
	Not interested/slightly	304 (13.8%)
	Missing	14 (0.6%)

There were no significant differences in perceptions of ambient AI scribes between primary care and specialty visits. Small but statistically significant differences were observed across gender, age, and race (Supplementary File). Perceived helpfulness differed by race (*P* = .001), with Asian respondents most likely to rate the tool as helpful (277, 86%), followed by Black (62, 78%) and White (1000, 74%) respondents (Cramer’s *V* = 0.10). Interest in future use varied slightly by gender: 749 (84.0%) of males preferred future use compared with 863 (76.1%) of females (*P* < .001). Interest in new technology was higher among males (*P* < .001), with smaller differences observed across race and age (*P* = .003 and *P* = .052, respectively). Patients who did not prefer future use were less likely to rate the tool as helpful (238/388, 61.3%; *P* < .001; *V* = 0.46) and less likely to report interest in technology (153/413, 37.1%; *P* < .001; *V* = 0.36) compared to those who preferred future use.

## Discussion

In an evaluation of patient perceptions of an ambient AI scribe integrated into clinical practice, the majority of patients who had experienced the technology found it acceptable. Most found the tool helpful and desired its use with future visits, with small demographic differences. Since most respondents were aged 70 years or older, these findings may reflect perceptions of ambient AI scribes in an older adult population. A potential limitation is selection bias, as patients who chose to respond to the post-visit survey may have had more positive attitudes toward technology. An additional limitation is that the survey denominator and response rate were not available. This study also did not specifically assess patient perceptions related to communication with their clinician, privacy, trust, or perceived accuracy. In addition, the survey did not examine why some patients found the tool less helpful or were less supportive of future use. Future qualitative work, including patient interviews, could provide deeper insight into these differences and inform patient-centered design and workflow improvements. Co-design approaches may also guide expansion of ambient AI beyond note generation to other patient-facing use cases such as draft instructions or visit summaries.

## Conclusion

In one of the largest evaluations of patient perceptions of ambient AI scribes, most patients found the tool helpful and desired the use of ambient AI scribes with future visits. Together with prior clinician-focused research, these results suggest that ambient AI scribes may support clinician documentation while being acceptable to many patients in clinical practice. As health systems scale these tools, ongoing evaluation that incorporates both patient and clinician perspectives will be essential to ensure inclusive, effective implementation.

## Supplementary Material

ooag104_Supplementary_Data

## Data Availability

The data underlying this article will be shared on reasonable request to the corresponding author.
