# Supplementary material for: Patient perspectives on clinicians’ use of ambient AI scribes
Source: JAMIA Open. 2026 Jun 12;9(3):ooag104. doi: 10.1093/jamiaopen/ooag104 (PMC13264434; doi:10.1093/jamiaopen/ooag104)
Supplement: ooag104_Supplementary_Data [file ooag104_supplementary_data.docx]

**Supplemental Table**

Preferred Future Use, Perceived Helpfulness, and Interest in Technology by Demographics

| **Demographic** | **n/N (%)** | **p value** | **Cramer’s V** |
| --- | --- | --- | --- |
| **Interest in Future Use** |  |  |  |
| **Gender** |  | <0.001 | 0.10 |
| Female | 863/1,134 (76.1) |  |  |
| Male | 749/892 (84.0) |  |  |
| **Age group** |  | 0.872 | 0.03 |
| <30 | 24/32 (75.0) |  |  |
| 30–39 | 63/77 (81.8) |  |  |
| 40–49 | 74/97 (76.3) |  |  |
| 50–59 | 154/189 (81.5) |  |  |
| 60–69 | 357/452 (79.0) |  |  |
| 70+ | 948/1,189 (79.7) |  |  |
| **Race/Ethnicity** |  | 0.580 | 0.04 |
| Asian | 265/324 (81.8) |  |  |
| Black/African American | 60/80 (75.0) |  |  |
| Declines to state | 36/45 (80.0) |  |  |
| Other/Unknown | 173/225 (76.9) |  |  |
| White | 1,083/1,359 (79.7) |  |  |
| **Perceived Helpfulness** |  |  |  |
| **Gender** |  | 0.170 | 0.03 |
| Female | 850/1,126 (75.5) |  |  |
| Male | 685/877 (78.1) |  |  |
| **Age group** |  | 0.945 | 0.02 |
| <30 | 25/32 (78.1) |  |  |
| 30–39 | 60/76 (79.0) |  |  |
| 40–49 | 72/99 (72.7) |  |  |
| 50–59 | 147/190 (77.4) |  |  |
| 60–69 | 341/443 (77.0) |  |  |
| 70+ | 898/1,173 (76.6) |  |  |
| **Race/Ethnicity** |  | 0.001 | 0.10 |
| Asian | 277/322 (86.0) |  |  |
| Black/African American | 62/80 (77.5) |  |  |
| Declines to state | 33/42 (78.6) |  |  |
| Other/Unknown | 169/221 (76.5) |  |  |
| White | 1,000/1,345 (74.4) |  |  |
| **Interest in Technology** |  |  |  |
| **Gender** |  | <0.001 | 0.09 |
| Female | 1,031/1,235 (83.5) |  |  |
| Male | 844/943 (89.5) |  |  |
| **Age group** |  | 0.052 | 0.07 |
| <30 | 25/33 (75.8) |  |  |
| 30–39 | 69/77 (89.6) |  |  |
| 40–49 | 93/101 (92.1) |  |  |
| 50–59 | 171/197 (86.8) |  |  |
| 60–69 | 420/475 (88.4) |  |  |
| 70+ | 1,106/1,305 (84.8) |  |  |
| **Race/Ethnicity** |  | 0.003 | 0.09 |
| Asian | 322/346 (93.1) |  |  |
| Black/African American | 74/90 (82.2) |  |  |
| Declines to state | 39/45 (86.7) |  |  |
| Other/Unknown | 203/239 (84.9) |  |  |
| White | 1,244/1,465 (84.9) |  |  |

Percentages reflect row percentages. P values are from chi-square tests. Cramer’s V is reported as a measure of effect size. Denominators vary across analyses due to missing data.
